# School and childcare center drinking water: Copper chemistry, health effects, occurrence, and remediation

**DOI:** 10.1002/aws2.1270

**Published:** 2022-03-17

**Authors:** Elizabeth Montagnino, Darren A. Lytle, Joan Rose, David Cwiertny, Andrew J. Whelton

**Affiliations:** ^1^ Lyles School of Civil Engineering Purdue University West Lafayette Indiana USA; ^2^ U.S. Environmental Protection Agency Cincinnati Ohio USA; ^3^ Michigan State University East Lansing Michigan USA; ^4^ Department of Civil and Environmental Engineering University of Iowa Iowa City Iowa USA; ^5^ Center for Health Effects of Environmental Contamination University of Iowa Iowa City Iowa USA; ^6^ Public Policy Center University of Iowa Iowa City Iowa USA; ^7^ Lyles School of Civil Engineering and Division of Environmental and Ecological Engineering Purdue University West Lafayette Indiana USA; ^8^ Center for Plumbing Safety Purdue University West Lafayette Indiana USA

**Keywords:** children, copper, health, plumbing, schools

## Abstract

The study goal was to better understand the risks of elevated copper levels at US schools and childcare centers. Copper health effects, chemistry, occurrence, and remediation actions were reviewed. Of the more than 98,000 schools and 500,000 childcare centers, only 0.2% had copper water testing data in the federal Safe Drinking Water Information System database. Of the facilities designated public water systems, about 13% had reported an exceedance. Schools that were not designated a public water system (PWS) also had exceedances. Few studies document levels in schools and childcare centers. Widely different sampling and remedial actions were reported. Flushing contaminated water was the most evaluated remedial action but was unreliable because copper quickly rebounded when flushing stopped. Building water treatment systems have been used, but some were not capable of making the water safe. The health risk was difficult to determine due to the limited occurrence data and lack of best management practice studies. A national drinking water testing campaign and field studies are recommended.

**Article Impact Statement**

Limited information exists regarding the safety of school and childcare center drinking water quality for copper.

## 1 INTRODUCTION

Hydration is critical to child development as children are fundamentally dependent on safe drinking water for cognitive and physical health. More susceptible to dehydration than adults, children depend on adults for hydration (Bar‐Or, 1998; Bar‐Or et al., 1980; D'Anci et al., 2006). Mild dehydration can affect cognitive development such as decreased visual attention and short–term memory (Benton & Burgess, 2009; D'Anci et al., 2006; Edmonds & Burford, 2009; Edmonds & Jeffes, 2009). To help ensure proper hydration, the US Department of Agriculture implemented the National School Lunch Program in 2011 and required participating schools to provide water at mealtime (section 9. [42 US code § 1758]) (US Department of Agriculture [USDA], 2011). In 2016, the Child and Adult Care Food Program was added to this program to require childcare centers to supply access to water throughout the day (section 17. [42 US code § 1766]). Methods of access include “water pitchers and cups on lunch tables, a water fountain or a faucet that allows students to fill their own bottles or cups with drinking water” (USDA, 2011). These programs do not require confirming that the water provided is safe to drink.

While much recent discussion has focused on lead in drinking water, little effort has been dedicated to copper. Copper can leach from copper‐containing plumbing materials posing acute and chronic health–related concerns to children. The federal health–based, maximum contaminant level goal (MCLG) for copper in drinking water is 1.3 mg/L, which is also referred to as the copper action level under the *Lead and Copper Rule* (LCR). The copper threshold however, unlike the lead action level, is based on *acute* health effects reported in Wyllie (1957) that include abominable pain, and gastrointestinal illness. A metallic or bitter taste can be detected at copper concentrations from 1 to 5 mg/L (Dietrich et al., 2004; Health Canada, 2018; Pizarro et al., 1999; World Health Organization, 2003, 2004). When concentrations are elevated (3 to 10 mg/L) the water may turn blue/green in color, depending on the particulate size of the copper by‐product deposited in the water (Barceloux & Barceloux, 1999; Edwards et al., 2000).

In the United States, school and childcare centers are either a customer of a public water system or the institution is themselves federally regulated because it produces drinking water from their own water supply source for more than 25 people more than 60 days per year (National Center for Education Statistics, 2016; USEPA, 2021a). Approximately 8,000 schools and childcare facilities (1.3% of the total in the United States) are public water systems regulated under the Safe Drinking Water Act (SDWA) (USEPA, 2021a). The USEPA estimated that all other school and childcare centers (98.7%), approximately 98,000 and 500,000 respectively, are not regulated under the SDWA and may or may not have conducted voluntary drinking water testing (USEPA, 2021a). There is no federal requirement for schools and childcare centers that do not own their own public water system (PWS), to conduct copper testing at all drinking water locations before the building is occupied and during the life of the building. Therefore, drinking water safety within schools and childcare centers remains unclear.

For the very few schools and childcare centers that own a PWS (i.e., they have an independent supply well) they must comply with the federal LCR requirements. These were initially promulgated in 1991 and are currently being revised (USEPA, 2021c). The regulation 40 CFR (Code of Federal Regulation, 2020) part 141 – *National Primary Drinking Water Regulations* subpart I (40 CFR §§ 141.80–141.86) is dedicated to the control of lead and copper in drinking water (USEPA, 1991; USEPA, 2021b; CDC, 2014). Schools and childcare centers designated as PWSs are categorized as a non–transient non–community water system. Required sampling for these PWS consists of a first–draw sample after 6 h of stagnation (not overnight or weekend stagnation), with a 1 L wide mouth bottle for copper. Sampling requirements will vary slightly based on service line and distribution system pipe material, size of population served, and access to sampling locations. The number of sampling locations is based on the population size and which locations are representative of the plumbing, and sampling locations, and should avoid point of use (POU) or point of entry (POE) treatment devices (USEPA, 2021b). Initial and routine lead and copper sampling occurs every 6 months. Water systems may reduce monitoring frequency (annual, triennial or every 9 years), based on 90th percentile sample results at or below the lead and copper action levels, population size, and water quality (Title 40 Code of Federal Regulations Section 141.86). If a system exceeds the copper or lead action level, the system must implement corrosion control treatment or source water treatment steps according to the rule. Though, unlike lead, copper does not require public education (i.e., notifying the patrons of the building of the exceedances).

The objective of this review was to better understand the current knowledge associated with copper in school and childcare center drinking water and provide recommendations for those organizations seeking to identify and address copper contamination in their buildings. Specific aims were to: (1) examine acute and chronic health impacts associated with the ingestion of copper contaminated water, (2) identify the factors that contribute to copper occurrence in schools and childcare center drinking water, (3) summarize school drinking water copper occurrence studies, and (4) examine the effectiveness of remedial actions to address copper in drinking water. While the health impacts of lead are well recognized and studied, impacts of copper ingestion are less discussed. Yet, growing awareness over building water safety and consideration of the copper's acute‐based exposure limit in water, particularly for children, has refocused the question of copper's health implications.

## 2 METHODS

The authors reviewed the peer–reviewed and gray literature, federal and state government resources, as well as reports produced by the US National Academies of Science, Engineering, and Mathematics (2000). Keywords used during the search included “copper,” “school,” “copper corrosion,” “drinking water,” and “copper health.” Databases used were primarily Web of Science and Google Scholar. Very few water testing studies were found for US schools, so the literature search was expanded to other countries. US federal and state regulations, as well as school water sampling results examined in this review were found on available local and state government websites. Other investigative efforts involved identifying published media reports regarding school water sampling results and industry POU and POE water treatment device literature. The SDWIS database of regulated water systems and contaminants for public water systems was searched for text that included “is school or daycare.” Then, those PWS were put into excel and an equation was used to search for key phrases likely to appear in the names of schools (“sch,” “elem,” “learn,” etc.). PWS's containing at least one of those phrases were identified as schools, while the rest were classified as daycares. The PWS that are listed as schools or daycares were found by using this search on July 7, 2020.

## 3 RESULTS AND DISCUSSION

### 3.1 High levels of copper can cause acute and chronic health impacts

Copper is an essential nutrient, but high intake of soluble copper salts is associated with gastrointestinal illness and liver toxicity in some susceptible individuals (Wyllie, 1957). Humans absorb copper through food and drinking water. When the intake of copper increases, the body's ability for absorption decreases. Both the intestinal tract and the liver are involved in copper metabolism and storage (Olivares et al., 2002; Turnlund et al., 1989). There are, however, some susceptible individuals that have a genetic condition that prevents the body from regulating excess copper and thus can develop a syndrome known at Wilson's disease which causes cirrhosis of the liver. The gene associated with this disease is found globally in humans at a frequency of 0.3 to 0.7% (Uauy et al., 2008).

Copper intake for dietary purposes has been estimated at between 0.45 and 1.2 mg for infants and adults, respectively (Pennington et al., 1989). According to the National Institutes of Health, the recommended dietary allowances are 0.44, 0.7, 0.89, and 0.9 mg for ages 4 to 8, 9 to 13, 14 to 18, and greater than 18 years of age, respectively, while the tolerable upper intake levels for these age groups are 3, 5, 8, and 10 mg, respectively (NIH, 2021). It was in the 1950s that the toxicity of copper associated with gastrointestinal illness, the most common symptom, was noted via an accidental ingestion (15 adult individuals) which was acute and immediate (copper doses were estimated to be of 5.3 to 32 mg) (Hopper & Adams, 1958; Wyllie, 1957). The 1.3 mg/L copper MCLG was determined by taking the lowest copper concentration reported by Wyllie (5.3 mg/L) that resulted in these symptoms. That sample was taken from a cocktail in a copper tumbler, that had been sitting in the refrigerator for about 2 h, resulting in elevated copper concentrations from dissolution. The 5.3 mg/L concentration was then divided by an uncertainty factor of 2 to achieve for 2.65 mg per 2 liters (L) (Fitzgerald, 1998; Knobeloch et al., 1994). This MCLG was decided to be at a level where no‐observed‐adverse‐health effects were noted, and achieved by using a 2‐fold safety factor and assuming 2 L of water consumed per day (Donohue, 1997; USEPA, 1991). Taylor et al. (2020) reviewed the health implications of copper ingestion, and some key findings are summarized in Table 1.

**TABLE 1:** Some health impacts of copper exposure by ingestion (adapted from Taylor et al., 2020)

Health impact shown with a single dose	Symptoms	Minimum levels	Population
Acute toxicity, accidental and experimental ingestion	Nausea most common (75%), others include diarrhea, vomiting (food or blood); Stomach pain; Black, “tarry” stools; headache; difficulty breathing; An irregular heartbeat	2.65 to 4 mg/L: lowest‐observed‐adverse‐effects level 2 mg/L: NOAEL	Human adults
Liver failure Neurotoxicity	Liver malfunction Dementia and anxiety	Toxic accumulation even at low levels, diagnosed at 8 to 50 years of age	Wilson's disease patients
Reproductive effects	Toxicity occurred at doses that also caused maternal toxicity	381 mg/L: NOAEL	Rats
Developmental	Maternal toxicity, reduced food intake, abortion, death	6 mg/kg body weight/day: NOAEL	Rabbits
Neurologic	Deficiency or excess of copper vaguely associated with neurodegenerative Alzheimer's disease (oxidative damage related to copper and other metal ions)	Drinking <0.01, 2, 4, or 6 mg/day in water for 2 months no effect found	(*n* = 48 men; 49 women)
Mutagenic, carcinogenic	No evidence of such impact	—	—

Abbreviation: NOAEL, no‐observed‐adverse‐effects level.

### 3.2 Limitations of documented waterborne disease outbreak reports

US Center for Disease Control and Prevention (CDC) reports of waterborne disease outbreaks associated with copper contaminated drinking water ranged from a single individual at home to 74 cases associated with a single restaurant in Vermont (CDC, 2019, 2021). Generically, it was often stated in the records that “outbreaks were similar, occurred after highly corrosive water entered the pipes, addressed by addressing corrosive water.” For some restaurant and school outbreaks excess copper was released from copper pipes due to backsiphonage of CO_2_ and carbonated water from soda machines. In 1993, 43 illnesses were identified at a hotel associated with “corrosive” water (CDC, 1993). New homes and renovated homes with copper piping were associated with 27 cases over 2 months, supporting what is known about new homes being susceptible to copper exposure through drinking water via new copper pipes. In 1997, a release of sulfuric acid at a water plant into the water distribution system caused copper release.

Unfortunately, outbreak records contain very little data on what happened and over what time frame (e.g., days). Since the 1997 to 1998 surveillance reporting of drinking waterborne diseases, the CDC provided a decreased amount of incident reports relative to previous years (CDC, 1998, 2000). The decrease in reporting was attributed to: (1) an assumption that most copper disease outbreaks from drinking water occur in a private home, thereby affecting a small number of people and not becoming apparent to public health officials; (2) chemical exposure through drinking water can be difficult to identify and associate; (3) the waterborne disease outbreak database is not commonly known or referenced; and (4) chemical poisonings may be difficult for physicians to diagnose. Interestingly, since 2005 there have been no reports of copper–related waterborne outbreaks, which may be due to the reasons listed above.

For schools specifically, although outbreaks were documented, the information available was limited. For example, the surveillance report published in 2001 only included a brief description of a school waterborne outbreak, noting that consumers mentioned the water was bluish in color and symptoms occurred quickly following consumption (CDC, 2004). No quantitative evidence was given. The outbreak was thought to be caused by an improperly installed anti‐scaling device (CDC, 2021).

The sparse information reported by the CDC over approximately 40 years makes it difficult to understand copper‐related waterborne disease outbreaks in the United States. As reported in Knobeloch et al. (1994), only a few studies have reported acute illnesses due to the ingestion of copper contaminated drinking water in the United States and beyond. One study of new and renovated homes in Sweden reported three children with chronic diarrhea and copper levels below the United States. MCLG of 0.22 to 1.1 mg/L. The symptoms occurred due to the copper exposure along with the lower weight of children. (Stenhammar, 1979). In Vermont, USA, a new house had levels between 2.8 and 7.8 mg/L, which was associated with residents having recurring vomiting and abdominal pain (Spitalny et al., 1984). In another study in Sweden, copper concentrations greater than 1 mg/L, were associated with vomiting, diarrhea, colic, and constipation for children (Pettersson & Kjellman, 1989). In Wisconsin, copper and unexplained diarrhea was investigated, and it was found that copper levels exceeding 3 mg/L (ranging from 0.09 to 12 mg/L) was likely a common cause (Knobeloch et al., 1994).

### 3.3 Sources and factors contributing to copper occurrences in schools

Copper in drinking water can originate from sourcing materials such as, piping, tanks, and valves (Barn et al., 2013; Lytle & Schock, 2000; Ra et al., 2020) and can even be added to control other contaminants such as *Legionella* in hot water systems (Liu et al., 1994; Miuetzner et al., 1997; Springston & Yocavitch, 2017; Stout et al., 1998; Stout & Victor, 2003). Even when plastic piping is used, copper can leach from brass valves and fixtures (Huang et al., 2019; Sarver & Edwards, 2011). Copper is susceptible to uniform, nonuniform (pitting), galvanic, erosion, and other forms of corrosion. As a result, “scale” made up of copper minerals and potentially other constituents can form on the inside wall of the pipe (Ferguson et al., 1996; MWH, 2005). Like lead release mechanisms associated with lead‐containing materials (Del Toral et al., 2013; Deshommes et al., 2017; Kim & Herrera, 2010; Lewis et al., 2017), this scale can be a source of particulate copper in the case of hydraulic sheering and other disturbances (Edwards & Jacobs, 2000). Copper can also dissolve into the water from scale due to changes in environmental conditions (Calle et al., 2007; Merkel et al., 2002).

**FIGURE 1:**
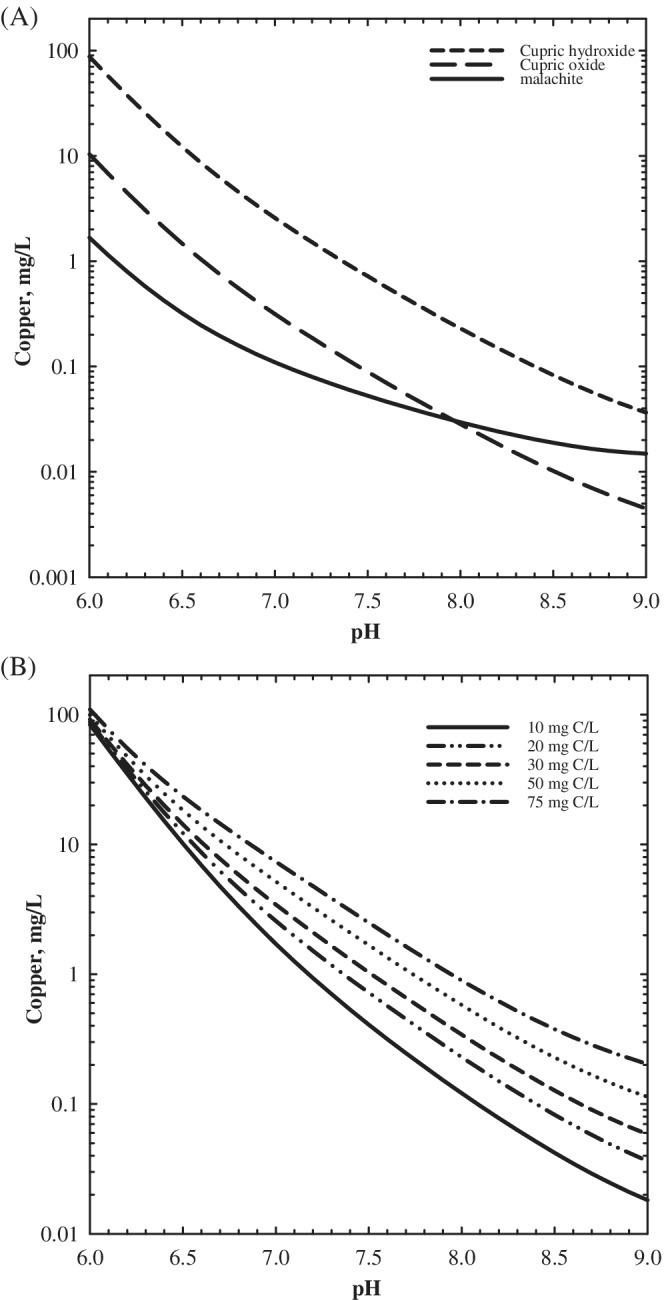
The impact of (a) “age” on Cu(II) mineralogy and solubility with pH (20 mg C/L dissolved inorganic carbon [DIC], *I* = 10 mM), and (b) pH and DIC on the solubility of “new” plumbing (assuming cupric hydroxide as the solubility‐controlling solid, *I* = 10 mM). Theoretical simulations were performed using R programming language based on previous work (Schock & Lytle, 1995a) and fundamental chemistry principles. Thermodynamic equilibrium model inputs were selected from past work: cupric hydroxide (Schindler et al., 1965), cupric oxide (Schindler et al., 1965), and malachite (Symes & Kester, 1984)

In oxic and disinfected drinking waters, Cu^2+^ [Cu(II)] mineralogy and chemistry largely controls copper release into the water (Ferguson et al., 1996; MWH, 2005). The relationship between Cu(II) solubility in tap water and important water quality parameters such as pH and dissolved inorganic carbon (DIC) are well established (Ferguson et al., 1996; Schock et al., 1995a, 1995b). For example, Cu(II) solubility decreases with increasing pH, and DIC complexes in new plumbing (i.e., cupric hydroxide assumed to control copper solubility) have been found to dominate copper speciation above pH 6.5, resulting in increased Cu(II) solubility with increasing DIC (Schock et al., 1995a; Figure 1a). Temperature also has an important role in copper solubility and water quality (Boulay & Edwards, 2001; Rushing & Edwards, 2004).

The prediction of copper levels in tap water is complicated by the reported aging phenomena of copper minerals. Over time (years to decades), scale and its mineralogy changes which can result in decreased Cu(II) solubility (Edwards et al., 2002; Lagos et al., 2001; Schock et al., 1995a; Turek et al., 2011). The cupric hydroxide model provides a conceptual understanding of copper solubility changes as Cu(II) solids age in water (Schock et al., 1995a). The model proposes that Cu(II) solubility–controlling solids on the surface of copper plumbing “age” from a relatively soluble copper solid to an insoluble one. The aging process of copper most likely involves the re‐crystallization and dehydration of relatively soluble copper hydroxide, Cu(OH)_2_, or possibly georgeite (Lytle et al., 2019), Cu_2_(CO_3_)(OH)_2_·6H_2_O, into less soluble tenorite, CuO, and the slow formation of malachite, Cu_2_CO_3_OH, in pH ranges below the general stability boundary of tenorite (Figure 1b). Given the importance of plumbing age on copper levels at the consumer's tap and water quality impact on Cu(II) solubility, worst case copper levels can be anticipated in relatively new homes and buildings plumbed with copper plumbing, particularly in elevated DIC and/or lower pH waters in the absence of orthophosphate corrosion control treatment.

Exactly how the copper aging process and total scale formation relate in terms of solubility transformation, water quality and frequency of use is not well understood but scale formation can progress over years to decades. Cuprosolvency can be reduced temporarily by the presence of sulfate, bicarbonate, and orthophosphate which can cause immediate changes to the type of solid present in water systems that hold cupric hydroxide or cupric ion solids. At lower pH values, adding orthophosphate rapidly decreases Cu(II) solubility, however, at higher pH values, adding orthophosphate could stabilize higher levels of copper corrosion, which could not be achieved without adding (Schock et al., 1995a; Schock & Sandvig, 2009). Edwards et al. (2002) demonstrated orthophosphate and polyphosphate treatment helped to reduce soluble copper on long time scales. The study also further confirmed that at certain pH values and DIC levels, orthophosphate interferes with the formation of other copper minerals that typically control solubility on the long term. Short‐term orthophosphate use decreases copper concentrations observed in some cases. This comparison illustrates the complexities involved in making copper phosphate solubility predictions (Lytle et al., 2018). These possible effects should be considered when trying to reduce the release of copper corrosion by‐products from building plumbing.

### 3.4 A clear knowledge gap: Occurrences in school drinking water

#### 3.4.1 Peer‐reviewed studies: Case studies, test results, and remediation

When the authors searched the Web of Science Database for the keywords “lead,” “school,” “drinking,” and “water,” 219 entries were found. When the keyword lead was changed to copper only 32 results were available, only approximately 14% of the total number of results when “lead” was used. Only 12% (4/32) of the results were school case studies associated with copper exposure. When the word “schools” was replaced with “childcare centers” no results were returned associated with “copper” but returned two for “lead.” Two other results returned for “lead” when “daycare” was substituted for “childcare centers.” Switching “childcare centers” to daycares did not return any results for “copper.” Peer‐reviewed studies which investigated and reported copper concentrations in school and childcare drinking water systems have been sparsely published over a period of just about 30 years since the *LCR* has been published. Peer‐reviewed studies were found only for a few schools in the United States: 50 by Murphy (1993) and 1 by Ra et al. (2020) representing 51 schools out of 98,000 in the United States. The two US school studies were 27 years apart. One peer‐reviewed drinking water testing effort of a single US childcare study was found (Grace et al., 2012). As a result of limited evidence available, the review was extended outside the United States but still only resulted in a few studies in Canada (Barn et al., 2013; Doré et al., 2018) and Saudi Arabia (Al‐Saleh, 1996). Another childcare center study was completed in Sweden, but was not translatable (Berg, 1981). These studies will be discussed in chronological order.

In the United States, the 1993 study examined copper in 50 schools in New Jersey, USA. (Murphy, 1993). Two water fountains per school were sampled at “first draw,” after a 10 min flush (i.e., running the tap to clear the building pipes of its current water and bring in fresh water from the water main.) and at lunchtime (just before the school's first lunch break, approximately 3 to 5 h after the first‐draw samples). It is unclear how many fountains per school existed however. The maximum copper levels for these sampling events were similar: (first draw maximum 10.2 mg/L), (10 min flush 7.8 mg/L), (before lunch break 8.5 mg/L). Median first draw levels (0.26 mg/L) were greater than the after flushing (0.068 mg/L). Though, by lunch time, the median copper level was greater at 0.12 mg/L. Murphy (1993) concluded that school building water systems should be flushed every 2 to 3 h and for 5 to 10 min to reduce copper levels. It is not clear why the 5 min minimum was chosen because the study only used 10 min flushes. Additional study limitations include the lack of description of the types of plumbing materials, volume of water to each fixture, fixture use frequency, and sampling location in relation to the POE device.

In Saudi Arabia, drinking water testing was conducted at 32 primary schools (Al‐Saleh, 1996), and most of the school buildings were rented households. Two high use drinking water fountains were tested at 27 schools and one water fountain was available to test at five schools for resulting in 59 water samples. The copper concentrations found had a range of 2.19 to 106.69 μg/L. Of the highest sample results, using the European Economic Community elevated copper guideline of 100 μg/L, the average copper level was 105.32 ± 1.94 μg/L. No remedial actions were discussed.

Two studies that summarize water testing and remedial actions at numerous schools in Canada were the most extensive studies found. From May 2012 to June 2012, water sampling was conducted in 11 schools at a total of 130 locations (Doré et al., 2018). The majority of service lines were lead; therefore, copper was assumed to originate from building plumbing components. For larger buildings without corrosion control the first draw copper concentration ranged from 141 to 619 μg/L compared to smaller buildings with corrosion control (13.7 to 63.5 μg/L) and without corrosion control (73.5 to 415 μg/L). Different fixture types, sampling protocols, and building sizes were considered. The investigators reported that concentrations differed between fixtures in the same building (up to 202‐fold) and varied (up to 39‐fold) at the same tap depending on sampling protocol and the abundance of particulate copper. The maximum copper level reported in the study was 1,566 μg/L, which was discovered at a water fountain. A 30 second flush decreased the copper level by 59% from the first draw sample but levels above California's Public Health Goal of 300 μg/L sited by the author persisted after flushing.

Four schools in Canada also underwent testing in 2014 (Barn et al., 2013). The investigation was initiated because salmon eggs unexpectedly died in a classroom aquarium. The subsequent study analyzed drinking water in both elementary and secondary schools because “water intake differs by age.” The most frequently tested school had nine fixtures sampled with pre‐ and post‐ flushing samples collected. The stagnation time before fixture sampling (pre‐flush) was not described. Fixtures sampled were water fountains, kitchen sinks, and breakroom sinks. The authors reported that the elevated copper levels originated from the building's plumbing. The investigators “estimated [that copper] intake far exceeded the daily [recommended daily allowance] for both elementary and secondary school aged children.” Flushing did not always reduce copper levels, sometimes causing levels to increase. The mean copper level before the flush (stagnation period not declared) in one elementary school ranged from 0.4 to 7.6 mg/L and was 0.05 to 10.7 mg/L after a 5 min flush. Overall, the mean value was reduced from pre‐flush 4.9 mg/L to post‐flush 2.0 mg/L. Though, at a secondary school, the mean pre‐ and 5 min flushed copper levels were 2.2 to 4.0 and 0.5 to 7.0 mg/L post‐flush, but the mean value increased from 3.2 to 4.3 mg/L, respectively. The degree of stagnation before the first‐draw sample was taken is unknown. Ten minutes of flushing was recommended to reduce copper levels below the aesthetic Drinking Water Guidelines, which is 1 mg/L (Health Canada, 2018). Remedial actions suggested to reduce the elevated copper levels were: (1) adjusting pH, (2) adding corrosion inhibitors; (3) removing lead and copper containing plumbing; (4) implementing a flushing protocol for highly stagnated fixtures, (5) limiting use to only cold water; (6) restricting use at outlets where there are elevated levels, (7) installing point of use filters, and (8) providing alternative sources of water such as bottled water.

An investigation reported on a childcare center with copper levels above the action limit located on Wright‐Patterson Air Force Base in Ohio (Grace et al., 2012). The study took place between October of 2005 and January of 2006 and investigated the effectiveness of orthophosphate as corrosion control in a new building with elevated copper levels associated with high alkalinity water. Additional studies investigating the role of stagnation on copper levels in the distribution system were conducted in September 2006. To understand the copper levels of the water supply before adding orthophosphate, samples were collected biweekly between October and December 2005 at five sampling locations in the building. These sampling locations were strategically chosen to create a complete profile of the building distribution system, with fixtures at varying distances from the point of entry. The following sampling protocol was implemented: First, the tap was flushed for 3 min and then a 250 ml sampled was collected. Next, the water sat stagnant for 12 h before another 250 ml sample was taken. The sampling before orthophosphate was added revealed that every copper sample after the 12 h of stagnation was above the action limit ranging from 1.4 to 2.4 mg/L. The samples (17) taken after the initial 3 min flush had copper levels ranging from 0.1 to 2.0 mg/L, with four flushed samples (4/17) having levels above the action limit. Orthophosphate was added to the system in January 2006 and copper levels remained above 1.3 mg/L at most locations until March 2006. The stagnation study done in September 2006 revealed that copper concentrations increased the most in the initial 10 h of stagnation, although the copper levels still increased beyond the 10 h. In one example, a sample taken after 12 h of stagnation was 0.8 mg/L and increased to 1.4 mg/L in the following 60 h, after a total of 72 h of stagnation. These results highlight the importance of sampling protocol and duration of stagnation, as the copper levels continued to increase above the action limit after the required 12 h of stagnation before sampling as also seen in Doré et al. (2018).

In 2018, an investigation was conducted at a seven‐year‐old Indiana US Green Building Council LEED certified school. Test results were interpreted considering building design, sampling, and remediation (Ra et al., 2020). Researchers discovered copper present in drinking water throughout the school during summer break, as well as after students and staff returned in the fall. Water fountains, kitchen sinks, as well as bathroom and laboratory sinks were sampled. Copper levels entering the school from the public water supplier were negligible, but a statistically significant relationship was found between copper concentration and the length of copper pipe between the building entry point and the sampling location. During the summer, 50% of the water outlets further inside the building often saw copper exceed 1.3 mg/L (27/54 samples). The maximum copper concentration reported was 2.72 mg/L at a cold water fixture. Once fall classes began, 37% of cold water taps still exceeded the 1.3 mg/L and the average cold water copper level decreased. The school‐wide mean cold‐water level during the summer (1.4 mg/L) decreased to 0.66 mg/L when returning in the fall. Interestingly, hot water sampled at the same location for the purposes of another study rarely exceeded 1.3 mg/L (2/54 samples) and did not contain disinfectant. In a prior bench‐scale study, copper level was not found to be influenced by water temperature (Dodrill & Edwards, 1995). Flushing water at faucets did not consistently reduce copper concentration, and sometimes copper levels were greater after flushing. The drinking water delivered to the school throughout the study had high DIC levels. No explicit plumbing code language was found that cautioned designers and builders about excessive copper leaching when in plumbing is exposed to high alkalinity water (IAPMO, 2020; ICC, 2018).

### 3.5 Government reported copper testing results

For the schools and childcare centers who have their own water source or treat water received from a neighboring water system (i.e., they are considered consecutive systems), they are likely designated as a PWS (depending on population served) and are required to comply with the LCR. Since 1992, 17,653 unique PWSs have been entered into SDWIS as schools and childcare centers (USEPA, 2021a, 2021b, 2021c). Based on PWS name, it was estimated that schools and childcare centers constituted 12,193 and 5,460 of the systems, respectively. Since 1992, a total of 6,419 copper action level exceedances have been reported by 2,332 different schools and childcare centers (13.2% of all schools and childcares in the database). Considering only schools and not childcare centers, 5,108 copper action level exceedances associated with 1,831 schools (15% of all schools in the database) were reported, since 1992. It seems there are a significant number of copper drinking water challenges as database records indicate.

Separately from PWS requirements, some school districts and state governments have established voluntarily testing for copper in drinking water (Table 2), often as part of concurrent lead testing programs and because common analytical approaches for lead can be easily adapted to monitor for copper in the same water sample. For example, the Detroit Department of Education, the Massachusetts Department of Environmental Protection, and Maryland's Carroll County Public School system tested for copper in school and childcare centers starting 2016. Alexandria County Public Schools in Virginia began testing for copper in 2019. Table 2 provides a summary of the copper found and remedial actions taken in these schools based upon their voluntary testing efforts. Studies indicated that water quality varied by regional and geographic location, building and pipe age, and level of education in the building tested (Alexander & Lewis, 2014; Hood et al., 2014; Patel et al., 2013).

**TABLE 2:** Schools and childcare centers with reported copper levels exceeding the 1.3 mg/L health‐based limit on voluntary testing efforts

State/year	No. of schools	No. of schools with copper levels above USEPA AL	Remedial actions	Reference
Detroit, Michigan/2016	133	11	Adding Corrosion Control. Water fountains with onsite filtration systems.	Detroit Department of Health (2016)
Massachusetts/2016	~2,000	Schools–344 Childcare Facilities–7	Remove fixtures from use. Flush for 1 min. Use cold water only.	MassDEP (2016)
Carrol County Public Schools, Maryland/2016	40	4	Signs were placed on the faucets still in use saying “Not for Drinking/Not for Potable Use.” Fixtures are to be replaced.	CCPS (2016)
Alexandria County Public Schools, Virginia/2019 and 2020	25	Winter 2020–6 Winter 2019–9	Remove the fixture from utility. Flush the lines (time unknown) and replace aerators. Retest, and if the issues persist, replace the fixtures or pipes.	ACPS (2020)

Abbreviations: AL, Action level; USEPA, US Environmental Protection Agency.

Approximately 2,000 Massachusetts schools and childcare facilities have participated in copper water testing, of which 2,302 out of the 84,153 fixtures (e.g., water fountains, kitchen sinks, and classroom sinks) tested were either greater than or equal to the 1.3 mg/L MCLG (maximum 53.2 mg/L at kitchen faucet). These results represent approximately 344 schools and 7 childcare centers with copper levels above 1.3 mg/L. The remedial actions implemented included flushing each tap for 1 min before use, posting “Do Not Use” signs on fixtures, and only using cold water taps (MassDEP, 2016).

Detroit, Michigan's Health Department supported copper testing at schools and childcare centers in 2016. For each of the 133 participating schools, three water samples were drawn, including high use water fountains and kitchen faucets. Of the schools, 11 found copper at more than 1.3 mg/L (maximum 15.5 mg/L at a kitchen faucet). As a remedy, Detroit Public Schools installed ion exchange systems at water fountains and added corrosion control to the main water system for a few school districts (Detroit Department of Health, 2016).

Carroll County Public Schools in Maryland participated in testing in late fall 2016 (November–December). Across 40 schools, the maximum level of copper, 10.2 mg/L, was found for a kitchen sink. The fixtures dedicated for drinking water were taken offline, whereas fixtures associated with non‐potable water (e.g., primarily water for cleaning) are still in use. Signs were placed on the faucets still in use saying, “Not for Drinking/Not for Potable Use.” All fixtures that exceeded any action level (AL) were placed on a list for replacement, with drinking water fixtures designated as a priority. After the fixtures were replaced, secondary testing was required (CCPS, 2016). As of January 2017, it was reported that the “failed” fixtures were removed and replaced at 16 of the schools with copper exceedances (Chappell, 2017). It is unclear if any of the replaced fixtures had both copper and lead AL exceedances or just an exceedance with one metal.

School water quality test results were reported by the Alexandria County Public Schools in Virginia. In the winter of 2019, 25 schools, childcare centers and academic buildings participated, and nine organizations had buildings in exceedance of 1.3 mg/L. The maximum copper level reported was 7.73 mg/L at a sink. In the winter of 2020, 6 of the same buildings underwent an additional round of testing, and 3 buildings had copper levels in exceedance of 1.3 mg/L (with a maximum of 11.4 mg/L at a sink). The remedial actions implemented by the school district included: (1) removing the fixtures from use; (2) flushing the lines (time unknown); and (3) replacing aerators. After each remedial action, fixtures were to be retested. If remedial actions like flushing and replacing aerators did not lower copper levels, then the entire fixture was replaced. Finally, if the problem persisted after fixture replacement, it was recommended for the pipes to be replaced (Alexandria County Public Schools [ACPS], 2019).

### 3.6 Conflicting beliefs and perceptions about copper in drinking water with limited data

There are conflicting datasets pertaining to beliefs and perceptions about school and childcare center water safety. Optimistic studies in the United States show 92.6% of middle and 90.4% of high school students believe the drinking water supplied at school was “clean or very clean” and only 11% of participants across the United States in another study believed that water is unsafe in schools (Hood et al., 2014; Long et al., 2018). Other studies are not as positive. Another 2014 US study found 40% of participants did not believe water fountains at school were “clean and safe,” and a study in California showed stakeholders expressed concerns about drinking water quality in schools and confusion over policy (Onufrak et al., 2014; Patel et al., 2010).

Challenges for managing copper contamination in school and childcare centers are likely because of mixed public and school staff beliefs about school water quality and access, confusion about what organization handles testing school drinking water, different plumbing codes, and persistent questions over sampling protocol (e.g., how to collect samples, appropriate stagnation periods, how often, and at what locations). According to a 2014 US study, in the *opinion* of the school's foodservice personnel, 86.4% of elementary schoolers, 87.4% of middle schoolers and 89.4% of high schoolers were enrolled in schools that “met drinking water requirements” but it seems water potability was assumed and water testing was not conducted (Hood et al., 2014). This result is inconsistent with an analysis of the 2014 data from results in a database managed by the CDC (Cradock et al., 2019). Of the schools surveyed in 2014, 92% were supplied by a municipal water source. About 50% of the schools supplied by municipal water sources voluntarily tested the drinking water for bacteria (51.4%), coliforms (48.5%), and other contaminants (48.9%). When the voluntary water quality test results were received, few schools (38.3%) communicated results to school faculty and staff, to students' families (22.7%), and students (15.8%) (Cradock et al., 2019). Only 46.5% of schools surveyed flushed after stagnation periods (to remove water with higher amounts of contaminants), 45.8% conducted routine testing for lead, and only 25.6% reported giving training to custodial and maintenance staff on school drinking water quality (Cradock et al., 2019). A 2016 study in Massachusetts found that only 59% of the Massachusetts schools surveyed met state plumbing codes (Kenney et al., 2016). Students may have less accessibility to drinking water if districts do not require a ratio of less than or equal to 100 water fountains per one student within adopted plumbing codes, although drinking water fountains are not the only source of drinking water, as potable water can be obtained from multiple locations in school buildings (e.g., cafeterias, outdoor spigots and bathroom faucets) (Onufrak et al., 2014). Evidence suggests work is needed to determine the degree beliefs relate to the actual state of school plumbing and water safety.

### 3.7 Strategies to consistently reduce copper levels in existing building water systems

To reduce the risk of children being exposed to copper contaminated water, a series of approaches have been reported in the literature, though their practicality and efficacy has gone largely unresearched. When elevated copper levels were found in several school districts, the fixtures were shutdown and some were replaced (ACPS, 2016; CCPS, 2016; MassDEP, 2016). Flushing the contaminated water from the plumbing and replacing it with fresh water has been recommended (Murphy, 1993). Water treatment device installation (e.g., ion exchange and filters) and corrosion control measures (i.e., corrosion control agent, chemical addition) have also been mentioned (Doré et al., 2018; Grace et al., 2012). Limited data exist to validate the effectiveness, economic consequences, and length of benefit of copper drinking water reduction actions.

Despite the fact that flushing can have other benefits such as the delivery of disinfectant residual to fixtures and removal of water with greater concentrations of bacteria, heavy metals, and other contaminants (Michigan Department of Environment, Great Lakes and Energy, 2018; Proctor et al., 2020; Rhoads et al., 2020; USEPA, 2020), more recent peer‐reviewed evidence indicates flushing should not be relied upon to remediate copper contamination. In the literature and in practice, flushing was the most often mentioned copper remediation approach (ACPS, 2020; Barn et al., 2013; Doré et al., 2018; Grace et al., 2012; MassDEP, 2016; Murphy, 1993; Ra et al., 2020; USEPA, 1991). The intent of flushing in a building would be primarily to remove copper particulate material and accelerate copper pipe scale age. Multiple studies reported that flushing outlets at a copper piped schools and childcare centers did not consistently reduce copper levels at the water outlets, and sometimes copper levels increased in response to flushing (Barn et al., 2013; Doré et al., 2018; Grace et al., 2012; Ra et al., 2020). Flushing requires the building owners to supply staffing with proper training. Approaches will also differ based on the building design and volume of water within the distribution system from service line to the furthest tap. Many persons and hours may be needed per building depending on the plumbing design (Ra et al., 2020), and rate of copper rebound in less than 30 min to levels above 1.3 mg/L may render the flushing activity meaningless or require additional flushing (Dore et al., 2018; Hood et al., 2014; Patel et al., 2010; Ra et al., 2020). Copper exceedances were often attributed to pipes and fittings throughout the building.

The use of POE and POU water treatment technologies have been successfully applied to reduce copper levels in schools, specifically ion exchange and reverse osmosis processes, but are not capable of addressing all copper problems. Importantly, POE and POU treatment processes require validation that they are working, continued testing, and proper maintenance to ensure they *continue* to perform as desired. Patel et al. (2010) noted that the use of POU devices can be costly to California schools. Currently, manufacturers will use industry certification for devices intended for commercial building water treatment. Some commercial devices pertaining to copper removal from drinking water, including ion exchange systems, are associated with NSF International Standard 53 and 58 certifications. The NSF International Standard 53 requires a copper reduction from 3 mg/L to below 1.3 mg/L (NSF international, 2016). Table 3 summarizes the testing conditions; the filters undergo a 16 h testing period, where water containing “3000 ppb copper” is pushed through the device to determine if the device reduces levels below “1300 ppb.” The product must be tested at the highest and constant flow rate. Notably, under real‐world conditions multiple metal ions may be present such as lead, dissolved and particulate copper, and dissolved or particulate organic matter, all of which may influence device performance. In some situations, these contaminants can compete for ion exchange adsorption sites and foul filtration surfaces. Water alkalinity, pH, hardness, temperature, and turbidity levels in real buildings can also differ from those which the devices are challenged against. For reverse osmosis treatment, the NSF International Standard 58 certification focuses on total dissolved solids (TDS) reduction, not copper. NSF International Standard 58 does not purport to achieve a health‐based reduction of copper like the NSF International Standard 53 (NSF International, 2016). POE and POU water treatment devices should be selected based on understanding the water characteristics of the water to be treated. Several of the school and childcare center water testing studies reviewed indicate copper levels in excess of 3 mg/L. Therefore, the NSF International certified POE and POU devices are not recognized to reduce copper levels at those buildings below the 1.3 mg/L health‐based limit. No certification standards were found that purport to confirm POE and POU devices can reduce copper to less than 1.3 mg/L when copper is in excess of 3 mg/L.

**TABLE 3:** Test water quality challenge criterion for copper reduction reported by a product testing laboratory compared to characteristics for natural waters and public water systems

Parameter	NSF internationaltest method		Drinkingwater, range	References
	Low pH	High pH		
Alkalinity (mg/L as CaCO_3_)	10 to 30	100 to 250	<50 to >400	USEPA (1983)
Hardness (mg/L as CaCO_3_)	10 to 30	100 to 250	0 to >300 mg/L	
pH	6.5 ± 0.25	8.5 ± 0.25	<4.3 to >10	Murray and Reeves (1977); Pieper et al. (2018)
Polyphosphate (mg/L as P)	<0.5	<0.5	<0.2 to >3	McNeil and Edwards et al. (2002)
TDS (mg/L)	< 100	200 to 500	<20 to >500	Whelton et al. (2007)
Temperature (°C)	20 ± 2.5	20 ± 2.5	<7 to >26	Salehi et al. (2020); Whelton et al. (2015)
Turbidity (NTU)	<1	<1	Data not found	—

Abbreviation: TDS, total dissolved solids.

In terms of addressing copper corrosion through an entire school, full‐scale water treatment options such as aeration and chemical addition are available and have reliably prevented copper exceedances of 1.3 mg/L, when maintained (Grace et al., 2012; Kirmeyer et al., 1994; Lytle & Schock, 1998; Lytle et al., 1998). It is expected that certain conditions will stand‐out as being aggressive toward copper: (1) low pH, low DIC water (typical of many New England ground water, for example), and (2) high DIC groundwater (typical of many Midwest ground water, for example). In the case of low pH and DIC source waters, whole school treatment options include basic chemical addition, aeration, and limestone contactors. Aeration is typically reserved for situations where the water has specific chemical composition. As said previously, copper solubility in water is dependent on pH and DIC, and DIC sources include, carbonic acid (H_2_CO_3_), bicarbonate ion (HCO_3_^−^) carbonate ion (CO_3_^2−^), and carbonate metal complexes (e.g., CaHCO_3_^+^). Dissolved carbon dioxide (CO_2_), theoretically equal to the H_2_CO_3_* concentration, decreases the pH and increases the corrosivity of the water. By aerating the water, CO_2_ content can be reduced (Edwards et al., 1996). Aeration as a method is favorable under conditions where the CO_2_ concentration is 4 to 10 mg/L when the pH is low (<7) and DIC concentration significant (>5 mg/L C). A case study at a New England 200‐person school used a diffused bubble aeration system and was able to reduce copper levels to below 1.3 mg/L (Lytle et al., 1998). Water from the bedrock well source was suitable for aeration (pH of 6.1 to 6.3, DIC 15 to 18 mg/L C and a CO_2_ concentration of 30 to 40 mg/L). At this school, aeration effectively reduced the DIC to 6 to 7 mg/L C and increased the pH to 7.47 ± 0.09, thereby reducing observed copper concentration. When a water is not chemically suitable for aeration, orthophosphate addition can be introduced. In the case of a childcare center in Ohio, orthophosphate was added at the building entry point, and this reduced copper levels throughout the building (Grace et al., 2012).

The acute illness caused by copper toxicity has a rapid onset, so it may be reasonable for public health professionals, in particular school nurses, to be educated about symptoms of water toxicity and how to sample. This would allow for rapid water sampling of sources the child has had accessed. This may include water fountains, sinks, kitchen faucets, other fixtures, and also water entering the building. Efforts could also be focused on preventing copper toxicity by monitoring water systems, and making school health officials aware of the monitoring and results. Schools that are designated as public water systems with multiple exceedances of the copper action level or those with high alkalinity water should be on alert in regard to the problems that could exist for the students. Schools that experience low occupancy or water use (e.g., during breaks, repairs, or shutdowns), should be aware that such conditions could aggravate copper release to the drinking water.

## 4 CONCLUSION

Children are more susceptible to acute and chronic copper toxicity than adults and ingestion of school drinking water poses a health risk. The literature review revealed that few studies have examined copper in school drinking water, including the health effects, occurrence, and chemistry making it difficult to fully understand the potential health risk and validate design and remediation options. Testing protocols and intervention approaches varied across studies. Available datasets indicate conflicting beliefs and perceptions about school drinking water safety. Evidence is clear that when a copper contamination problem is found, there is discrepancy between what actions are recommended. Observations in the peer‐review literature indicating flushing should not be relied upon. Flushing and water treatment installations require labor and funding making the goal of long‐term remediation difficult to study. POU/POE device use also has limitations; observed copper levels have exceeded the performance criteria for certified treatment systems. Furthermore, while a PWS can be in compliance with federal law, the building itself may prompt water to accumulate unsafe levels of copper.

The lack of copper testing for new buildings and plumbing design also merits attention. Certain waters (i.e., high alkalinity) may prompt greater copper leaching than others, and thus copper and copper‐containing plumbing components may be more susceptible to corrosion. Plumbing codes lack explicit consideration of high alkalinity water and copper corrosion. Copper levels may be negligible once water enters the building, but water use, environmental conditions, and the plumbing's design may prompt in‐building exceedances. If, POU or POE devices are in use it is necessary to provide maintenance and periodic testing, as if not properly serviced, health risks can emerge. Capital and reoccurring costs associated with the devices should be considered. It is likely higher levels of copper in school and childcare center drinking water may exist at less frequently used fixtures, after holidays, and where there is low building occupancy, though previously undetected widespread building contamination can also exist as shown in the literature. Evidence suggests copper occurrence in schools and childcare centers merits more attention through a national drinking water testing campaign due to significant issues with previous studies, varied sampling protocols, children being susceptible, and only limited data available.

## CONFLICT OF INTEREST

The authors do not declare any conflicts of interest.

## AUTHOR CONTRIBUTIONS

**Elizabeth Montagnino:** Conceptualization; data curation; formal analysis; investigation; writing – original draft; writing – review and editing. **Darren A. Lytle:** Conceptualization; resources; data curation; formal analysis; validation; investigation; methodology; writing – review and editing. **Joan Rose:** Conceptualization; resources; data curation; formal analysis; validation; investigation; methodology; writing – review and editing. **David Cwiertny:** Data curation; methodology; writing – original draft; writing – review and editing. **Andrew J. Whelton:** Conceptualization; resources; data curation; formal analysis; supervision; funding acquisition; validation; investigation; visualization; methodology; writing – original draft; project administration; writing – review and editing.

## Data Availability

The data that support the findings of this study are available from the corresponding author upon reasonable request.
